# Acute effects of radiation treatment to submental muscles on burrowing and swallowing behaviors in a rat model

**DOI:** 10.1371/journal.pone.0268457

**Published:** 2022-05-13

**Authors:** Suzanne N. King, Evan Greenwell, Nada Kaissieh, Lekha Devara, Zachary Carter, James Fox, Megan Blackburn

**Affiliations:** 1 Department of Otolaryngology–Head and Neck Surgery and Communicative Disorders, School of Medicine, University of Louisville, Louisville, Kentucky, United States of America; 2 School of Medicine, University of Louisville, Louisville, Kentucky, United States of America; 3 Department of Radiation Oncology, University of Louisville, Louisville, Kentucky, United States of America; University of Colorado School of Medicine, UNITED STATES

## Abstract

Swallowing impairments are a major complication of radiation treatment for oropharyngeal cancers, influencing oral intake and quality of life. The timing and functional consequences of radiation treatment on the swallowing process is not clearly understood. A rodent radiation injury model was used to investigate the onset of oral and pharyngeal dysfunctions in deglutition related to radiation treatment. This study tested the hypothesis that (Wall et al., 2013) alterations in normal biting, licking, and swallowing performance would be measurable following 64Gy of fractionated radiation to the submental muscles; and (Kotz et al., 2004) radiation will affect the animal’s general well-being as measured via burrowing activity. Seven rats received radiation using a clinical linear accelerator given in 8 fractions of 8Gy and another seven animals received sham anesthesia only treatment. Swallowing bolus transit/size was assessed via videofluoroscopy, tongue movement during drinking was measured via an electrical lick sensor, and biting was analyzed from acoustic recordings of a vermicelli pasta test. Burrowing activity was measured by the amount of gravel substrate displaced within a container. Measurements were taken at baseline, during treatment (1–4 weeks), and after completion of treatment (weeks 5 & 6). Decreases in licking frequency and increases in inter-lick interval were observed 5- and 6-weeks post-treatment. Significant decreases in burrowing performance, swallowing frequency, and inter-swallow interval were observed starting the last week of treatment and continuing up to 2-weeks after completion. Results suggest that tongue dysfunction is one of the first treatment related feeding problems to present immediately after the completion of radiation to the submental muscles.

## Introduction

Dysphagia is a significant comorbidity of radiation-based treatments for oropharyngeal cancer. The most frequently observed swallowing problems result in alterations in bolus movement and bolus clearance [1]. These deficits can lead to long-term complications such as aspiration pneumonia, dietary restrictions, and malnutrition that negatively affect the quality of life of cancer survivors [2–5]. Radiation-induced dysphagia is designated into either acute or late complications based on the time of onset of clinical signs/symptoms [6]; however, evidence suggests that late onset dysphagia may be a consequential effect of acute toxicities and/or progressive changes that initiate during treatment [7]. The kinematic features of radiation-induced dysphagia are commonly identified in the clinic likely after muscle fibrosis and atrophy have progressed due to disuse and there are significant deficits in oral feeding at play. This is relatively late in the pathophysiologic process. Further insight into the timing and functional consequences of radiation are needed to understand the onset of swallowing problems and the motor behaviors affected by treatment to the submental muscles. This will help to identify mechanisms underlying swallowing dysfunction that may be beneficial to dysphagia rehabilitation planning.

The biomechanics of the oral and pharyngeal stages of swallowing require displacement of the jaw, tongue and hyolaryngeal structures to transport food/liquid safely from the oral cavity to the esophagus. These swallowing movements are controlled by a neural pattern-generating circuitry in the brainstem, which relies heavily on proper sensory feedback from the periphery to initiate and adjust to environmental conditions [8]. Previous work has indicated that the submental muscles consisting of the anterior digastric, mylohyoid, and geniohyoid are at high risk for radiation-associated impairments in swallowing function [9, 10]. Contraction of the submental muscles attribute to hyolaryngeal excursion and tongue base retraction, as well as oral motor functions i.e. jaw opening [11, 12]. Given the coordination between swallow-related muscles mediated by peripheral nerves and the brainstem [13], an injury to only the submental muscles could significantly disrupt the function of other muscles, even if they are not mechanically linked to the submental muscles [14]. Therefore, further work is needed to ascertain what swallowing functions are affected by radiation injury to the submental muscles. Clinical work has suggested that the onset of late radiation related swallowing problems begin during the course of treatment and progressively deteriorate over time [7, 15]. Van der Laan et al found that acute changes in eating behaviors (i.e. unable to eat solids/puree) and acute xerostomia during the last 3 weeks of radiation treatment were prognostic factors for swallowing dysfunctions occurring 6-months after treatment in head and neck cancer patients [7]. A study by Van den Steen et al examining eating function over the course of radiation treatment demonstrated that self-perceived swallowing function, functional oral intake, and tongue strength decreased 3-weeks into radiotherapy and persisted one-week after completion of treatment [15]. Wang et al also demonstrated that self-perceived swallowing function worsened between the third and seventh week of radiation treatment and correlated with weight loss and a decline in nutritional status [16]. Together, these studies demonstrate early disruptions in feeding abilities with radiotherapy, but it is unclear what swallow-related functions are most affected during and immediately after the course of treatment.

Rodent radiation muscle injury models have shown reductions in tongue force as early as 2-weeks after 35Gy of radiation (7Gy x 5) to the submental region [17]. Persistent changes in tongue strength have been observed at 3-months post treatment, along with declines in tongue muscle contraction time [18], and reductions in tongue displacement [17]. Similarly, studies have demonstrated changes in swallowing function two-weeks after 48Gy fractionated radiation to the mylohyoid muscle in the rat, including decreases in bolus transit through the pharynx and reduced pharyngeal bolus clearance during swallowing [19]. Further work is needed to ascertain when these alterations to swallowing kinematics initially present in the rodent model.

The objective of this study was to determine the onset of oral and pharyngeal swallowing dysfunction in response to radiation treatment in the rat. We studied functions involving both the oral and pharyngeal stages of swallowing as both phases have been implicated in radiation-induced dysphagia [20]. Previous studies have indicated that there is integration between these phases, and alterations in oral processing could affect how the bolus moves through the pharynx. We hypothesized that 64Gy of fractionated radiation focused on the mylohyoid muscle would alter normal licking, biting and swallowing behaviors starting at the end of radiation treatment. We also analyzed changes in the overall well-being of the animals during treatment using measures of burrowing performance. Although declines in physical quality of life measures is commonly seen clinically in patients receiving radiation [21], the impact of treatment-related functional decline has not been well-characterized in animal radiation-induced dysphagia models. We hypothesized that radiation treatment will reduce burrowing performance compared to baseline measures.

## Material and methods

Sprague Dawley adult male rats (12 months of age; 400-450g; N = 14) were used in these experiments. Male rats were chosen because the incidence of oropharyngeal cancer is higher in men than in women. Seven rats received radiation treatment, and the remaining seven rats were used as sham controls. Sham irradiation involved anesthetization and immobilization for a similar length of time as the radiation group. Animals were assigned into each group based on their burrowing performance. Rats with higher burrowing scores underwent radiation treatment. This method of assigning animals allowed us to use rats that were capable of burrowing to test the second hypothesis that overall well-being is affected by radiation. Burrowing is a rudimentary behavior and baseline scores signify that the rat can perform the task and are not indicative of a specific activity or stress level. Animals were housed two per cage under a 12:12 hour dark/light cycle where 6am to 6pm was dark and animals were most active.

All experimental protocols were approved by the Institutional Animal Care and Use Committee of the University of Louisville. All treatments and surgeries were performed under isoflurane anesthesia, and all efforts were made to minimize suffering.

Studying the onset of dysphagia is challenging in humans. Instrumental swallowing studies, which are necessary for accurately identifying swallowing impairments, are not regularly performed before or during the course of treatment. Further, clinical studies typically rely on indirect assessments (i.e. feeding tube duration, swallow-related quality of life measures, or other medical complications) to quantify changes in swallowing associated with radiation treatment. Rats provide a suitable model system to objectively assess motor dysfunctions that contribute to the pathophysiology of dysphagia following radiation treatment.

### Radiation treatment

Rats underwent irradiation using a Clinac 23iX (Varian Medical Systems, Palo Alto, CA, USA) as described previously [19, 22]. Rodents were anesthetized with isoflurane (2–4%) and placed in supine position. Six MeV electrons were applied and a 0.5cm layer of Superflab (CNMC, Nashville, TN) was laid over the outer surface of the skin above the submental space. This limited the maximum dose in the tissue to penetrate ~1.3–1.5 cm from the surface of the Superflab bolus. A 6x6 cm electron applicator with a standard 4x4 cm insert was used to collimate the electron beam. A 0.3 cm lead shield was placed anterior to the superflab material to further collimate the beam to the dimensions of the mylohyoid muscle (~11x 10mm). The radiation field size was held constant for each animal. Submental muscles were exposed to a total of 64Gy of radiation given in 8 fractions of 8Gy across roughly 4-weeks. The total dose was chosen based on clinical evidence indicating that doses between 50-70Gy to submental muscles are associated with high risks of radiation-induced dysphagia [10, 23, 24]. Treatment was given Monday, Wednesday, and Friday. All rats (irradiated and controls) received a 2-week break between the 3^rd^ and 4^th^ fractionated doses due to an unplanned shutdown with the instrument. The unplanned break between treatments might influence mucosal irritation due to the utilization of a hypofractionated radiation scheme. Topical aloe vera was given as skin treatment to reduce these symptoms. Weight loss was monitored daily and measured by the percentage of decline from initial weight.

Behavior testing was performed across multiple time points and on different days: baseline (day 0), during radiation treatment (weeks 1–4), and after the completion of radiation treatment (weeks 5–6). Reported time points are based on the initial start of radiation. For example, week 1 is one week after the first fraction of radiation. All trainings and behavior testing were performed during the animal’s dark phase when they are most active.

### Burrowing performance

To determine changes in overall well-being of the animals during radiation treatment, burrowing was examined as previously described [25, 26]. A burrow tube was made with a 3.5-inch PVC pipe with two steel screws placed on one end to elevate the open-end of the tube off the ground. A PVC end cap was used to close off the other end of the pipe. The tube was then filled with 2.5kg of pea-sized rocks (2-5mm) and placed in an empty cage without floor bedding. To minimize distractions from new smells during testing, each animal pair had their own burrowing tubes, rocks and cage. Prior to collecting baseline data, rats underwent ten training sessions as recommended previously to acclimate them to the experimental setup [25, 27, 28]. For the experimental condition, a single rat was placed into each cage-burrow setup for 60 minutes. There was no access to food or water in order to prevent distractions. The weight of the burrowing tubes was recorded before and after each session to measure the weight of rocks displaced from the tube. After the training period, animals with the highest baseline burrowing values were assigned to the radiation group. All irradiated rats exceeded the minimum recommended burrowing threshold at baseline of at least 500g [26]. This ensured that all the irradiated rats were able to burrow at baseline. Burrowing performance was assessed at baseline and once a week from 1–6 weeks after starting radiation or sham treatment.

### Videofluoroscopic swallow study

To analyze changes in bolus transit and bolus size during swallowing, rats were given chocolate milk (TruMoo, El Paso, Tx) mixed with 40% w/v barium sulfate ad libitum as previously described [14]. Animals underwent training sessions to acclimate them to the experimental conditions prior to testing. Drinking behaviors were recorded in the lateral plan using a GE Innova Model 3100 fluoroscope at a rate of 30 frames per second and magnification of 12 cm. Frame rate employed was the highest available for the fluoroscopy instrument used. During each session, individual rats were placed in a custom-designed cage as described above and were given 5 minutes to self-drink. Ten to twelve video clips at ~15 seconds in duration were recorded in each animal throughout the session during notable periods of drinking.

Video files were exported in DICOM format and relabeled using numeric codes. Videos were first viewed via ImageJ (NIH, Bethesda, MD) to identify video clips with more than three sequential swallows per animal. Of these, 3–5 separate video clips per animal were chosen randomly for analysis. Twenty swallows for each rat at each time point were measured and averaged. The drinking cannula (10mm) within the field of view was used as a calibration marker to scale images for size analysis. The second cervical vertebra (C2) was used to denote the end of the pharyngeal swallowing phase. This landmark was chosen because it is easily visible in all rats. The following VFSS metrics were calculated as previously described [14, 29, 30]: bolus speed through the pharynx, swallow frequency, inter-swallow interval, and bolus area within the pharynx. The onset of the pharyngeal phase was identified by a rapid movement of the bolus posteriorly towards the pharynx, which indicates inversion of the epiglottis. Pharyngeal transit time was determined by calculating the number of frames between the onset of the pharyngeal swallow to where the bolus passes C2 and then converted to seconds based on the 30 frames per second video frame rate. To determine bolus speed through the pharynx, pharyngeal transit time was divided by the calculated distance between the vallecula and C2 vertebra. Swallow frequency was quantified by counting the number of sequential swallows that occurred during the duration of time between the initiation of the first swallow cycle to the end of the last swallow cycle in the video clip. Inter-swallow interval was calculated by measuring the time between two sequential, uninterrupted swallows starting from the onset frame of the first swallow to the onset frame of the subsequent second swallow. Bolus size was calculated by outlining the bolus area in the pharynx (after the onset of pharyngeal swallow) and the known diameter of the fluid spout was used to scale the image from pixels to mm^2^. Two trained raters evaluated the VFSS measures. Interrater reliability was evaluated using intraclass correlation coefficient (ICC) of the data from at least 20% of the animals. ICC >0.90 was found, indicating substantial agreement across raters [31].

### Licking

To determine changes in the pattern of rhythmic tongue movements with radiation, licking behaviors were recorded using an electrical lick sensor as previously described [14]. During each session, rats were placed in a custom-designed cage with a spout located at the opening in the sidewall of the chamber. Chocolate milk was delivered through a metal spout with wiring wrapped around it that connected to the PowerLab system (ADInstruments, CO). To complete the electrical circuit, the grounding wire was connected to aluminum foil that covered the bottom of the cage. A positive voltage signal was generated whenever the rat’s tongue contacted the spout. The sides of the spout were covered with hard plastic tubing to limit contact of the spout to only the tongue during drinking. Distance of the spout to the rat was kept constant throughout the study to limit environmental influences on licking rhythm. Animals were given 10-minutes to drink after 3 hours of water deprivation. Rats underwent ten training sessions prior to collecting baseline data where they were introduced to the testing condition. Detection of licking events was performed offline using Spike2 software. The following licking variables were measured from the first 5-minutes of the drinking session once the animal began drinking: lick frequency, interlick interval (ILI), contact duration, total number of licks, and total number of clusters [32–36]. Licking frequency was calculated by counting the total number of licks during episodes of licking. ILI is the period between two consecutive licks and is calculated by taking the difference between the onset times of two successive licking cycles. Only ILIs <500ms were included in analysis as they represent natural pauses during ingestion when the tongue is moving anterior/posterior or medial/lateral toward the spout [32, 33]. Longer pauses with ILI’s >500ms occur when licking ceases and the animal moves their head away from the spout to perform an unrelated behavior (i.e. scratching, sniffing, etc). Lick contact duration corresponds to the time interval when the rat’s tongue is touching the spout. It is calculated by taking the difference between the rise and fall time of each signal above the threshold setting. The total number of licks per session was quantified within a 5-minute window of time. The number of clusters within the 5-minute window of time was also examined, as it corresponds to the amount of liquid ingested during testing. A licking cluster is defined as a period of uninterrupted licking until a pause of 500ms or more.

### Biting function

To evaluate gnawing performance in response to radiation treatment, pasta biting was tested using previously published methods [37, 38]. Each rat was given 10 pieces of 7cm long pasta at one time, in their home cage. Three hours before testing, food was removed from the home cage. Biting behavior was audio recorded using a condenser microphone suspended above the home cage. The acoustic signal was pre-amplified and recorded via Focusrite Scarlett 2i2 audio interface and Pro-Tools LE software (Avid) at a sampling rate of 44.1kHz and 24-bit depth. Preamplification settings remained constant throughout testing and background noise was monitored using a sound level meter. To confirm biting episodes, animals were video recorded using a Sony 4K HD video recorder. Semi-automated analysis of each peak was conducted via Spike 2 software (CED, Cambridge, UK). The acoustic signal was first converted into waveform, high pass filtered at 3kHz [39], and an intensity threshold line was set to separate biting events from background noise. The following variables were extracted from the data set: bite frequency, bite intensity, total number of bites, total biting time and the number of pasta pieces consumed [37–39]. Bite frequency was calculated by counting the total number of bites during periods of mastication. Biting intensity is an indicator of biting strength [37] and was measured by the amplitude of the biting signal. The total number of bites and total biting time were quantified during biting events within a 10-minute window of time.

### Statistical analysis

A one-way repeated measure ANOVA was conducted to determine if burrowing performance had an effect over time with radiation treatment. Bonferroni post hoc tests were conducted when warranted. Separate two-way mixed ANOVAs were performed to determine the impact of time and treatment on each swallowing, licking, and biting measure. Levene’s test showed that inter swallow interval and several licking variables had unequal variances and transformation of the data was unsuccessful. Since the group sizes were equal in this study and only mild departures from homogeneity of variance were observed, an ANOVA is still a validated option. When significant interactions were detected (p<0.05), univariant or repeated measures were used to compare groups and time with Bonferroni post hoc testing. When there was no significant interaction, but the main effects for group or time was significant then Bonferroni post hoc tests were performed. Data are the mean ± standard error. Statistical interpretations were made using IBM SPSS 27 (IBM Corp; Armonk, NY).

## Results

### Effects of radiation on weight

No substantial changes in weight loss were observed during or immediately after treatment with any of the irradiated or sham animals compared to weight at baseline. During radiation treatment, the rats body weight decreased by less than 5% from baseline. After radiation treatment, the average weight loss was 3% (standard deviation (SD) = 0.04). Couple days after starting sham treatment, the control animals had an initial weight loss of 2% from baseline. However, all the weight was regained 1-week after starting treatment and by the end of treatment they gained around 2% of their body weight relative to baseline (SD = 0.03).

### Decline in burrowing performance

**Fig 1:**
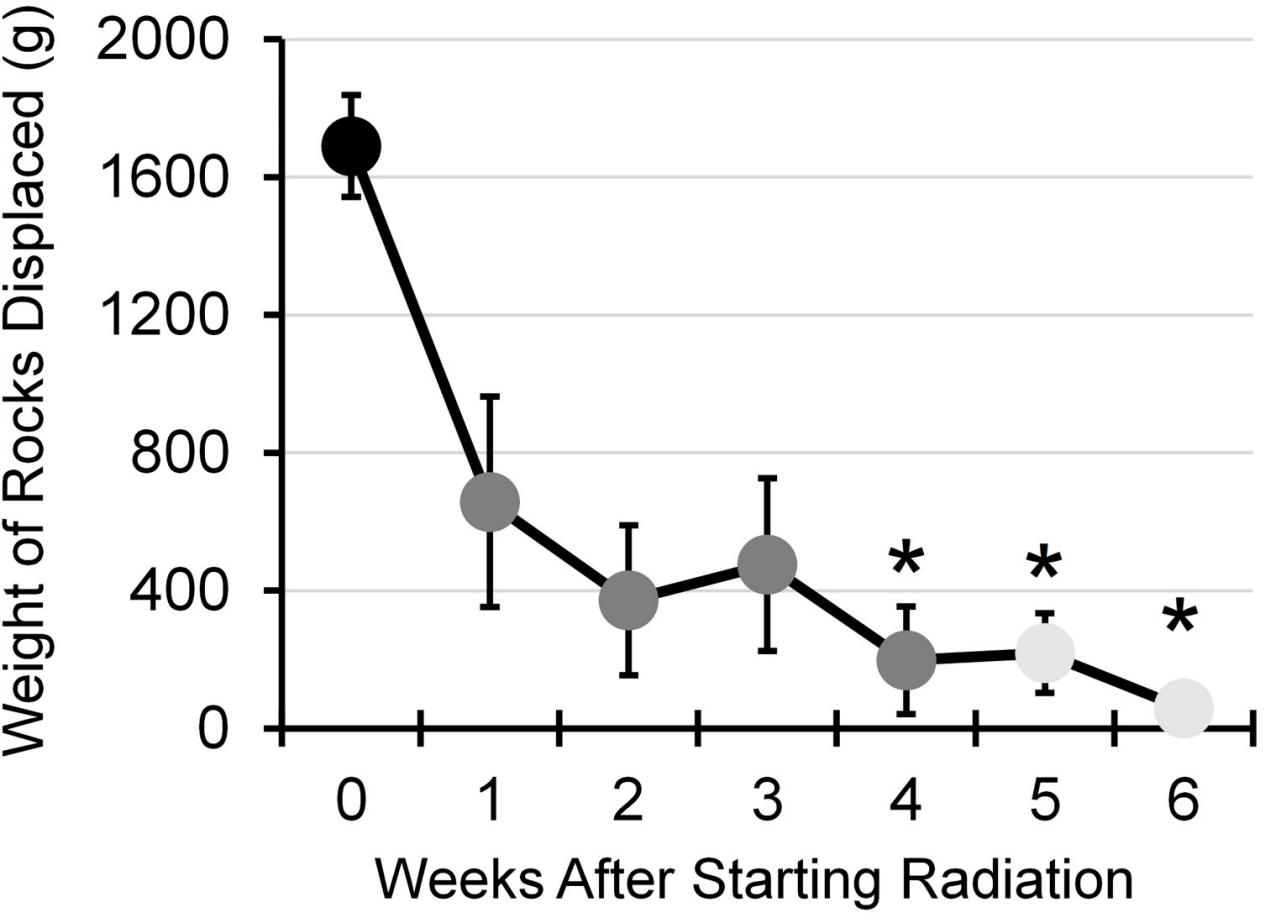
Burrowing behavior after radiation treatment. The graph shows the time course of change in the mean weight of rocks displaced. Data was analyzed using a repeated measures ANOVA to compare baseline (0; black), during treatment (1–4 weeks; grey), and after treatment (5 & 6 weeks; light grey). An asterisk indicates a statistical significance of p< 0.05 compared to baseline.

To determine if radiation treatment affected the animal’s overall well-being, burrowing activity was analyzed over the course of treatment. An ANOVA test was run to determine if there were changes over time. Radiation treatment elicited statistically significant differences in burrowing performance over time, *F*(6, 36) = 7.839, *p* < .001, partial η^2^ = .57 (Fig 1). Post hoc analysis with a Bonferroni adjustment revealed that burrowing behavior was significantly suppressed at 4-weeks (p<0.03), 5-weeks (p<0.02), and 6-weeks (p = 0.001) after starting radiation treatment compared to baseline. No significant changes were observed between baseline and 1–3 weeks after starting radiation.

### Alterations in swallowing function following radiation

**Fig 2:**
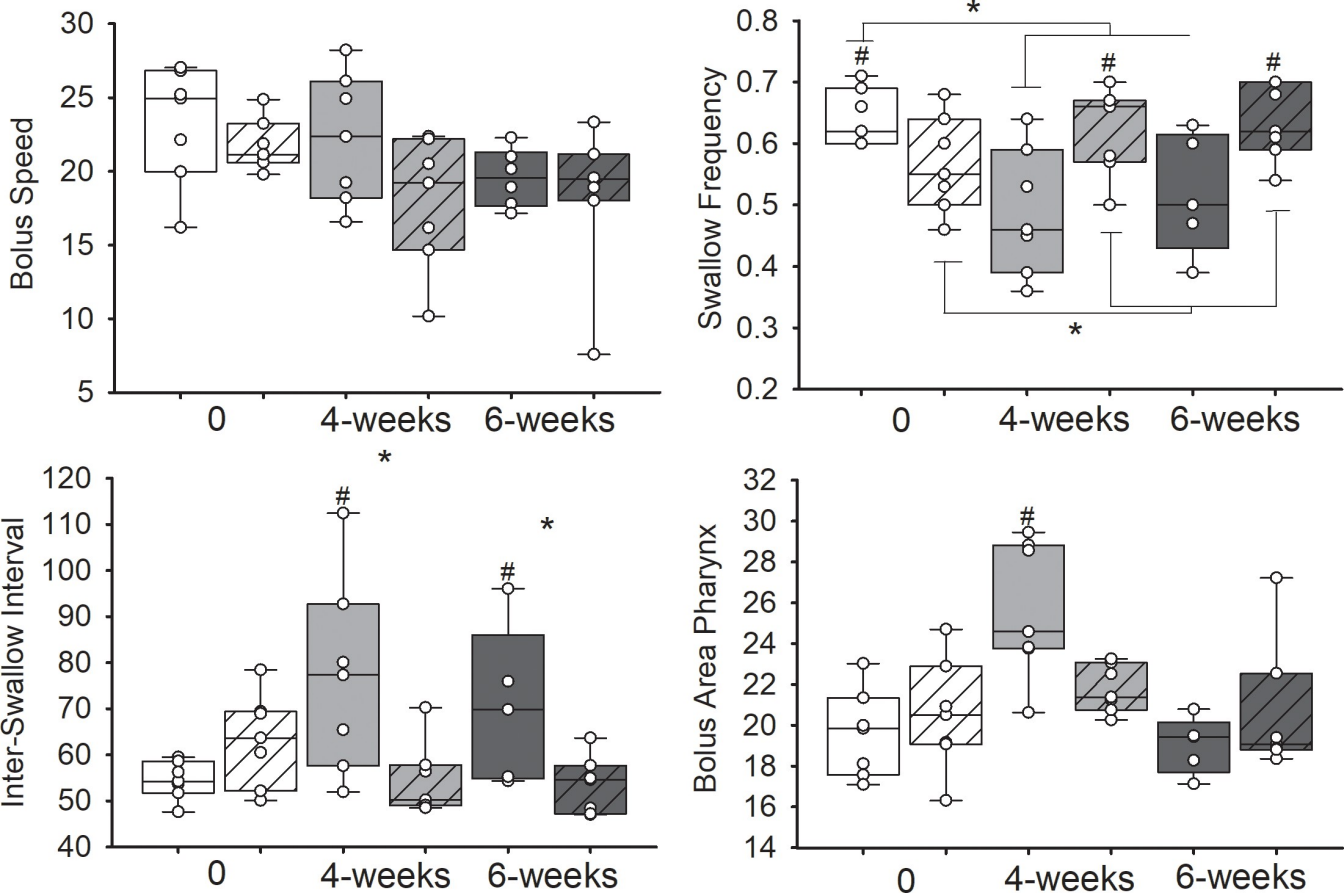
Changes in swallowing metrics taken from videofluoroscopic swallow studies. Radiated (no fill pattern) and sham (line pattern) treated animals were analyzed at baseline (0; white), at the end of treatment (4-weeks; grey), and after treatment (6-weeks; dark grey). Results demonstrate the mean and standard deviations for bolus speed through pharynx (mm/sec), swallow frequency (hz), inter-swallow interval (seconds), and bolus area in the pharynx (mm). The boundary of the box depicts the interquartile range and the line within the box marks the median. Whiskers above and below the box indicate the 90^th^ and 10^th^ percentiles. Each circle represents the mean value for a single animal. Statistically significant differences (p < 0.05) between groups are indicated by # and differences across time are designated by an asterisk.

To determine the effects of radiation on swallowing function, bolus transit patterns and bolus size during swallowing were analyzed at 0-, 4-, and 6-weeks after starting radiation. There was a statistically significant interaction effect between treatment and time on swallow frequency (F(2, 20) = 14.07, p< .001), inter-swallow interval (F(2, 20) = 9.10, p = .002), and bolus area in the pharynx (F(2, 20) = 6.45, p = .007) (Fig 2; Table 1). Post hoc testing revealed that the irradiated group had a higher swallowing frequency at baseline compared to the sham group (p< 0.05). By 4- and 6-weeks after the start of treatment, swallowing frequency significantly declined in irradiated rats compared to sham controls (p = .016; p = .029). Additionally, the time between swallows significantly increased at 4- and 6-weeks after starting radiation compared to controls based on increases found with inter-swallow interval (p = .022; p = .035). Significant increases in bolus size in pharynx was observed at 4-weeks after radiation compared to controls (p = 0.013). No significant group differences were found with bolus speed through the pharynx.

**Table 1:** Descriptive and statistically significant differences for swallowing metrics.

Measures	Treatment				p values				
		Baseline	4 weeks	6 weeks	Treatment x time effect	Rad vs Control at 4 wks	Rad vs Control at 6 wks	4 wks vs baseline	6 wks vs baseline
Swallow Frequency (Hz)	Radiation	0.63± 0.02	0.49±0.06	0.52±0.04	0.000	0.016	0.029	0.039	0.029
	Control	0.57±0.03	0.62±0.02	0.63±0.02				0.043	0.028
Inter-Swallow Interval (sec)	Radiation	56.5±1.1	78.9±11.2	70.3±7.7	0.002	0.022	0.035	—	—
	Control	63.3±3.8	54.5±2.9	53.4±2.3				0.013	0.014
Bolus Area (mm)	Radiation	18.5±0.6	26.4±1.7	19.0±0.62	0.007	—	—	0.007	—
	Control	20.5±1.0	21.8±0.4	20.6±1.2				—	—

Mean ± SE values.

To determine the simple main effects for time with the irradiated group, a repeated measures ANOVA was performed. Post hoc testing showed a significant decrease in swallowing frequency 4-weeks (p = .039) and 6-weeks (p = .029) after radiation compared baseline. The opposite was found with the control group, where there were increases in swallow frequency at 4- and 6-weeks after sham treatment compared to baseline (p = .043; p = .028). In the sham group, the time between swallows decreased from baseline to 4- (p = .013) and 6-weeks (p = .014) after starting treatment. Within the radiation group an increase in bolus size was observed in the pharynx 4-weeks after radiation compared to baseline (p = .007) and 6-weeks post radiation (p = .005).

### Changes in licking behavior following radiation treatment

**Fig 3:**
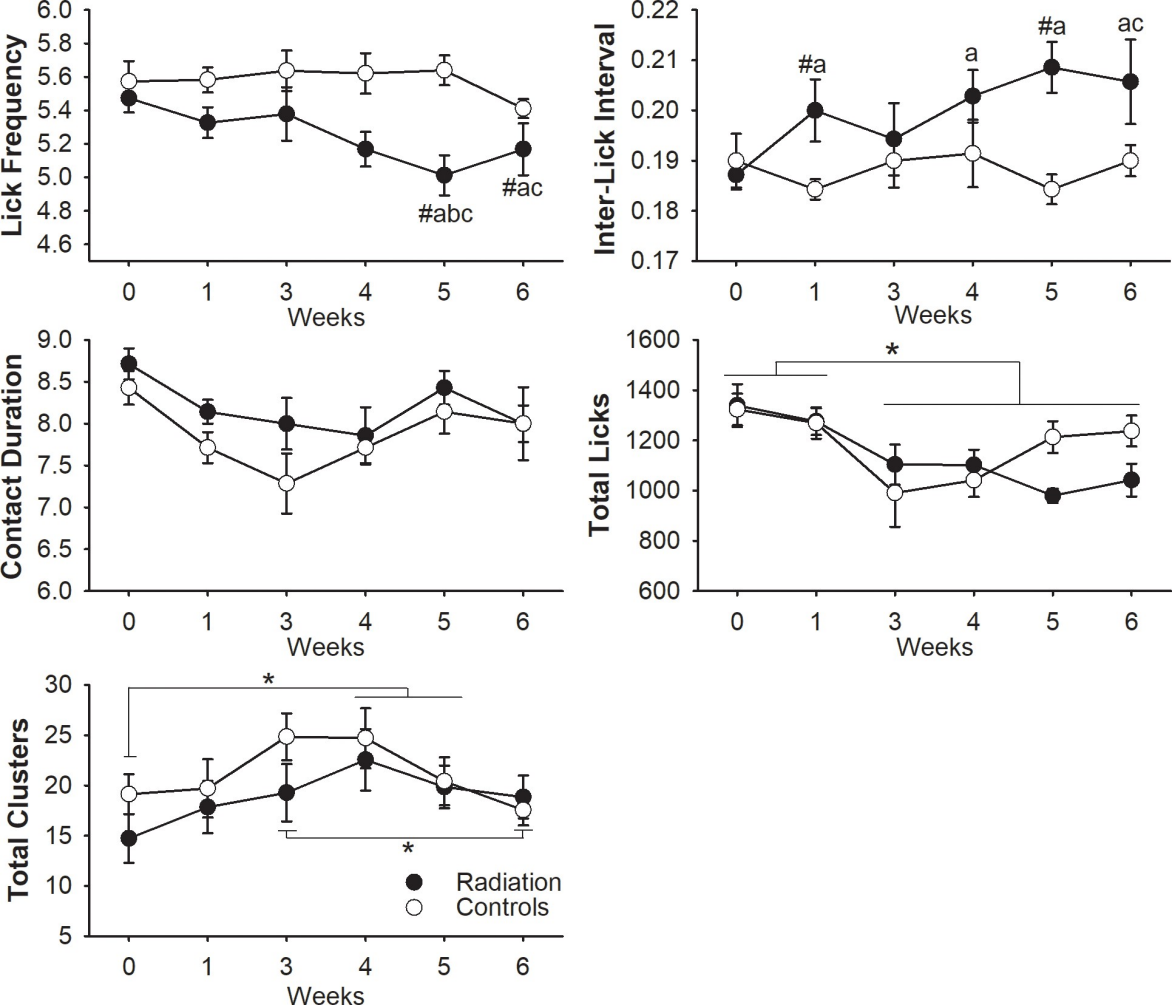
Alterations in the licking microstructure of rats treated with radiation. Licking was analyzed at baseline (0 [a]), during radiation (1- [b], 3- [c], and 4- [d] weeks), and after completion of radiation (black) or sham (white) treatment (5- [e] and 6- [f] weeks). Results demonstrate the mean and standard deviations for lick frequency (Hz), interlick interval (seconds), contact duration (seconds), and the total number of licks and clusters. Significant differences (p < 0.05) between groups are indicated by # and differences across time are designated by letters. Significant main effect for time was indicated by asterisks.

To determine the effects of radiation on the licking pattern, drinking behavior was analyzed at baseline and from 3–6 weeks after starting radiation treatment. There was a significant interaction effect between treatment and time in measurements of licking frequency (F(5, 60) = 2.36, p = .049) and interlick interval (F(5, 60) = 2.453, p = .043)(Fig 3; Table 2). Post hoc testing revealed at 4- and 5-weeks, the mean licking frequency decreased in irradiated rodents (p = .015; p = .001) compared to sham treated. At 1- and 5-weeks, irradiated rats exhibited a longer pause between sequential licks as indicated by an increase in interlick interval (p = .032; p = .001) compared to sham controls.

**Table 2:** Descriptive statistics for each treatment across time.

Measures	Treatment	Baseline (a)	1 week (b)	3 weeks (c)	4 weeks (d)	5 weeks (e)	6 weeks (f)
Lick Frequency (Hz)	Radiation	5.47±0.22	5.32±0.24	5.37±0.42	5.16±0.27*	5.01±0.31^abc*^	5.16±0.40^ac^
	Control	5.57±0.32	5.58±0.19	5.63±0.32	5.62±0.31	5.64±0.23	5.41±0.14
Inter-Lick Interval (sec)	Radiation	0.18±0.01	0.2±0.02^a*^	0.19±0.02	0.20±0.01^a^	0.21±0.01^a*^	0.21±0.02^ac^
	Control	0.19±0.01	0.18±0.01	0.19±0.01	0.19±0.02	0.18±0.01	0.19±0.01
Contact Duration (sec)	Radiation	0.09±0.01	0.08±0.01	0.08±0.01	0.08±0.01	0.08±0.01	0.08±0.01
	Control	0.08±0.01	0.08±0.01	0.07±0.01	0.08±0.01	0.08±0.01	0.08±0.01
Total Licks	Radiation	1339±226	1275±141	1104±212	1103±160	980±75	1042±172
	Control	1323±167	1269±166	991±356	1042±175	1214±167	1238±162

Mean ± SE values. Statistically significant differences (p < 0.05) between groups are indicated by an asterisk and differences across time are designated by a letter (a,b,c,etc).

Within the radiated group, significant decreases in licking frequency were observed at weeks 5 (p = .018) and 6 (p = .025) compared to pre-treatment (Table 3). Significant decreases were also found in licking frequency at 5- and 6-weeks compared to 3-weeks (p = .045; p = .046) after starting radiation, as well as decreases 5-weeks post-radiation compared to 1-week post (p = .05). In addition, significant increases in interlick interval were observed with the radiation group at 1-week (p = .035), 4-weeks (p = .005), 5-weeks (p = .003), and 6-weeks (p = .045) compared to baseline. Increases in interlick interval were also found between 3- and 6-weeks (p = .047) post. No significant differences in licking variables were found across time with the sham group.

**Table 3:** Statistically significant differences in the time points post treatment compared to baseline.

Measures	Treatment	p values					
		Treatment x time effect	1 week	3 weeks	4 weeks	5 weeks	6 weeks
Lick Frequency (Hz)	Radiation	0.049	—	—	—	0.018	0.025
	Control		—	—	—	—	—
Inter-Lick Interval (sec)	Radiation	0.043	0.035	—	0.005	0.003	0.045
	Control		—	—	—	—	—

There was no significant interaction effect on the total number of licks taken per session, but there was a significant main effect for time (*F*(5, 60) = 5.462, *p* < 0.005). Pairwise comparisons found decreases in the total licks after the start of treatment compared to baseline (3 weeks: p<0.02, 4-weeks: p<0.01, 5-weeks: p<0.01, 6-weeks: p<0.05) and 1-week post (3-weeks: p<0.03, 4-weeks: p<0.01, 5-weeks: p<0.001, 6-weeks: p<0.001). Main effects of time were also found with the number of licking clusters taken (*F*(5, 60) = 2.547, *p* < 0.037) with significant increases at 4- (p = .023) and 5-weeks (p = .043) compared to baseline and significant decreases at 6-weeks compared to 3- (p = .020) and 4-weeks (p = .036). No main effects for treatment group were found with total licks and total clusters.

### Biting function

To determine changes in biting behavior during and after radiation treatment, the acoustic signals from the pasta-biting test were analyzed. Measures were taken at baseline and once a week from 3 to 6 weeks after starting radiation treatment. Irradiated rats consumed all ten pasta pieces within the 10-minute period for each week tested; with exception, one rat 6-weeks after the start of radiation consumed only 7 out of 10 pasta pieces given. Sham treated rats consumed all 10 pasta pieces from 0 to 3 weeks after starting treatment. One sham treated rat at 4-weeks ate 9 out of 10 pieces; three out of seven control rats at 5-weeks consumed an average of 8.17 pieces of pasta. Animals typically picked up all the pasta pieces at once and then consume them either one at a time or dabble across each piece. Rats would perform biting episodes, which involved ~50 sequential bites, and then they take a break (> 3 seconds) before the next episode. Based on video analysis, these biting events appeared to have no relation to the pasta pieces; thus, rats took pauses in biting randomly, irrespective of how much of the remaining pasta piece they had left to consume.

**Fig 4:**
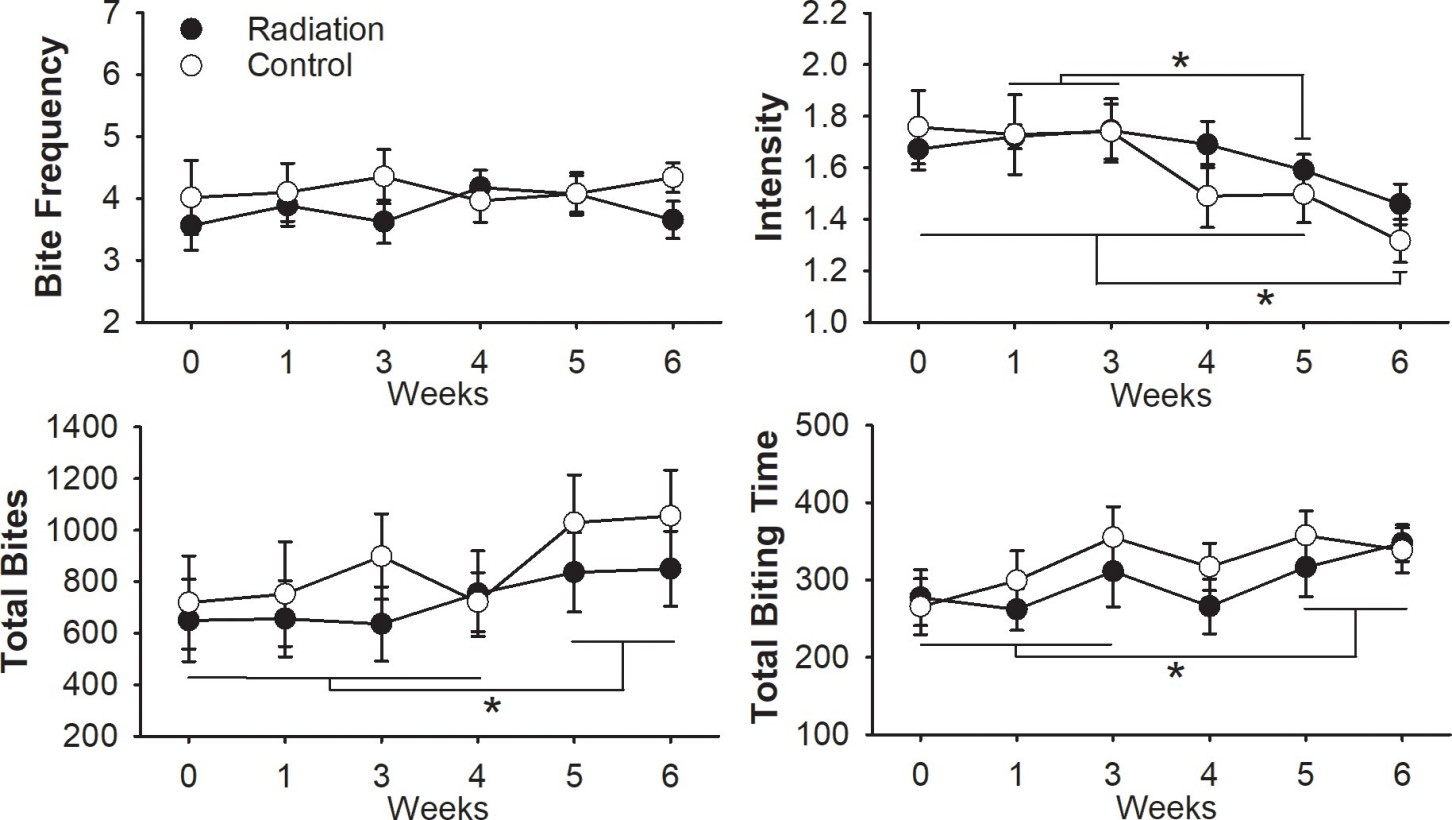
Changes in biting performance across time taken from the pasta biting tests. Biting was analyzed at baseline (0), during radiation (1-, 3-, and 4-weeks), and after completion of radiation (black) or sham (white) treatment (5- and 6-weeks). Results demonstrate the mean and standard deviations for bite frequency (Hz), intensity (dB), total bits, and the total biting time (seconds). No differences (p > 0.05) between groups were observed. Significant main effect for time was indicated by asterisks.

There was not a significant interaction effect between treatment and time on the biting variables tested (Fig 4). The main effects of treatment also showed no differences between groups. The main effect of time showed a statistically significant difference in mean biting intensity (*F*(5,60) = 7.383, *p* < .001, partial η^2^ = .381), total bites (*F*(3.15,37.82) = 10.015, *p* < .001, partial η^2^ = .455), and total biting time (*F*(2.71,32.55) = 3.623, *p* < .027, partial η^2^ = .232) at the different time points. Biting intensity was significantly reduced 5-weeks after treatment (radiation and control) compared to 1- (p<0.05) and 3-weeks (p<0.01). Six weeks after starting treatment, biting intensity significantly decreased compared to baseline (p< 0.001), 1- (p<0.001), 3- (p<0.001), 4- (p<0.03), and 5-weeks (p<0.03). Total number of bites increased at 5- and 6-weeks after starting treatment compared to baseline (p< 0.001), and 1- (p< 0.001), 3- (p< 0.05), and 4-weeks (p< 0.01). There was a significant increase in the total time biting at 5- and 6- weeks post compared to 0- (p< 0.05; p<0.01), 1- (p<0.02) and 3- (p<0.005; p<0.02) weeks after starting radiation.

## Discussion

The goal of this study was to identify oral and pharyngeal abnormalities in deglutition that occur during or immediately after radiation treatment in the rat and define when these changes begin. Although clinically, radiation is typically delivered concurrently with chemotherapy, there is a need to study each component individually to understand how they contribute to altering the model pathway. Radiation doses were administered at 8Gy in 8 fractions, which was tolerable in the rat as no adverse changes (i.e. weight loss) during treatment were found. Results showed that radiation impaired burrowing, licking, and swallowing function starting at the end of treatment, which appears to mimic the temporal course of the human condition.

Results suggest that impairments in tongue function were the most consistent treatment related swallowing problem observed with our radiation rat model. This is clinically significant due to the critical role the tongue plays in the oral and pharyngeal stages of swallowing including bolus formation and bolus propulsion from the oral cavity to the pharynx. Reductions in the timing and/or strength of tongue base retraction can produce insufficient bolus propulsion forces, which results in retention of the bolus in the vallecula or pharynx after swallowing. Decreases in swallow frequency and increases in inter-swallow interval were observed with VFSS after radiation treatment. During drinking, licking is periodically interrupted by a pharyngeal swallow once the fluid volume in the vallecula meets a certain threshold. Swallow frequency in this study accounts for the time it takes to draw in the fluid to the oral cavity via licking, trigger epiglottic retroflexion, and base of tongue retraction that moves the bolus downward through the pharynx. Thus, alterations in either oral or pharyngeal phases of swallowing can affect this measure. No measurable changes in bolus speed through the pharynx during swallowing were evident, but reductions in licking frequency during drinking were observed with the electrical lick sensor in irradiated rats at 5- and 6-weeks after initiation of treatment. Thus, the change in swallowing frequency and inter-swallow interval were likely due to prolongations in tongue movement during drinking in the irradiated animals. Our results are consistent with human studies that have shown impairments in tongue function and strength following radiation-based treatments [40, 41]. Clinical reports have demonstrated poor tongue control and decreases in motor strength 1-month following chemoradiation treatment [42]. Reduced base of tongue retraction during swallowing has been shown at 1–3 months after treatment, affecting 55–100% of patients with swallowing motility disorders after radiation-based treatments [43, 44]. Russell et al. also reported similar deficits in lingual strength 2-weeks and 5-months after 22Gy of total radiation treatment to the genioglossus muscles of the rat [18].

Fluid licking in the rodent is a rhythmic motor task where the tongue protrudes and retracts from the spout. To understand the aberrancies in tongue motor coordination that are contributing to decreases in licking frequency, we further analyzed changes in the temporal pattern during licking using data from the electrical lick sensor. Normal rats are thought to have a highly stable lick rate ~6-7Hz and modulate their fluid volume intake by either changing the total time spent drinking or the total number of licks performed [32]. In the current study, both of these measures remained constant between treatments and thus, there was no indication that fluid volume ingested affected licking frequency. One and two weeks after the completion of treatment, irradiated rats exhibited increases in interlick interval, which reflects the time between sequential licks from when the tongue retracts from the drinking spout moving the liquid bolus into the vallecula to when the tongue protrudes to re-contact the spout. Given that there were no measurable changes observed in the contact duration between the tongue and the spout, results suggests that radiation of the submental muscles influences tongue displacement during drinking. Interestingly, a previous study investigating changes in tongue strength in humans during the course of radiation observed significant decreases in posterior driving forces of the tongue starting at the fifth week of treatment, but no changes in anterior tongue strength were observed [15]. These differences were said to be related to regional differences in dosimetric parameters since patients included in their study received high total doses of radiation to the base of the tongue and pharyngeal muscles. However, recent work has shown that the submental muscles are critical for generating tongue base retraction during swallowing [12]. Specifically, Orsbon et al showed that posterior displacement of the tongue is produced through a piston-like mechanism involving contraction of submental muscles to elevate the floor of mouth and the hyoid bone, which reduces the posterior oral cavity volume. Therefore, radiating the submental muscles could alter the retraction and/or depression/elevation of the tongue. It is also possible that radiation injury provokes sensorimotor impairments (i.e. nerve injury) that would complicate discrimination of swallowing dysfunction based on the muscle(s) function alone [19]. Further animal work is needed comparing swallowing function in response to radiation to different swallow-related organs of interest (e.g. submental versus pharyngeal muscles).

In our study, no treatment related changes in biting function were observed. There is a physiological link between jaw and tongue movement [45], which supported further examination into the effects of this behavior. The biting phase is where the incisors come together via mandibular closure to separate particles of food and transport them posterior via the tongue. With our model, the digastric (anterior), mylohyoid, and geniohyoid muscles received the largest volume of radiation, albeit at slightly different doses. These muscles assist in depressing the jaw. The primary jaw closing muscles i.e. masseter, temporalis, and pterygoid were not directly within the radiation field. This may explain why there were no differences in biting variables between groups. Previous work in a radiation-induced oral mucositis mouse model found similar findings with no differences in biting force or gnawing time after radiation to the oral cavity [46]. In the current study, there was a significant main effect with differences in biting function between time. Specifically, we found decreases in biting intensity, increases in total biting time, and increases in total number of bites after treatment compared to baseline while the number of pasta pieces consumed remained stable across time. These results indicate that animals adjusted their biting strategy throughout the experiment. There are a few possible explanations for this result. Assuming that the biting variables do not change in non-treated, normal rats, then it is possible that the repeated exposure to anesthesia alone affected biting function. Other possible reasons for these changes in biting may be that the level of difficulty of the biting task was too easy, resulting in a ceiling effect or there was a learning effect where the experience of previous trainings and testing influenced subsequent biting tests.

Animal models provide insight into the mechanisms underlying radiation-induced dysphagia and can be useful in testing candidate drugs and predicting their responses. It is crucial for clinical translation that the animal model being studied be able to accurately mimic the human condition. In patients undergoing cancer treatment, weight loss is commonly observed and is an unfavorable indicator of poor prognosis during radiation [47]. Decreases are indicative of a decline in nutritional status and/or activity level [48]. Earlier studies using a similar rodent radiation model have not found significant changes in weight loss (>10%) during treatment with any animal tested [19, 49]. The increase in high-calorie food consumption that is required in training and performing various deglutition-oriented tests (i.e. videofluoroscopy and licking) may influence body weight measures. It is also possible that radiation alone does not affect an animal’s weight. The burrowing test was investigated for its feasibility in assessing the general condition of the animal during radiation therapy to determine if the animals experienced similar declines in activity as the human condition. We observed declines in burrowing function starting the last week of treatment and proceeding for 2-weeks after radiation ended. Rodents are well-known burrowers that perform this behavior for shelter and defensive purposes. Burrowing is thought to be sensitive to changes in the animal’s well-being and influenced by factors, such as motivation, anxiety, stress, pain, and depression [25, 26]. Therefore, results suggest that radiation treatment can negatively influence a rat’s quality of life and daily activities.

There are a couple possible causes for the acute changes in swallowing function. One possibility is that it is related to oxidative stress and/or DNA damage to the muscle that is known to be upregulated by radiation. In the diaphragm, a single dose of 10Gy of radiation has been shown to increase oxidative stress and decrease the viability of myocytes within 5 to 7 days post treatment [50]. Similar findings have been reported with fractionated radiation (4Gy x 4) to the rat hindlimb muscles, showing evidence of oxidative stress and mitochondrial dysfunction in the muscle up to 1-week post treatment [51]. Previous work has also demonstrated increases in transforming growth factor(TGF)-β gene expression in the mylohyoid muscle 2-weeks after 48Gy of fractioned radiation in the rat [49]. TGF-β can induce reactive oxygen species and modulate other cellular functions. These proinflammatory changes can damage the muscle and modify their fiber type resulting in mechanical problems. More work is needed in this area to understand the intrinsic changes in swallowing-related muscles after radiation treatment.

There are technical limitations that warrant further discussion. We were not able to analyze burrowing function in the sham group and rule out an anesthesia only effect because a portion of our rats did not meet the recommended burrowing threshold at baseline. Secondly, the frame rate used for VFSS was not ideal as rats swallow at a faster rate. Thus, small differences in swallowing metrics may not have been observed in this study. Lastly, although the total radiation dose used (64Gy) falls into the normal-high dose range given to patients treated for oropharyngeal cancers, high doses per fractionation were administered. Although this improved feasibility, it could cause increases in inflammation at the mucosa.

In summary, results suggest that tongue dysfunction is one of the first measurable changes in deglutition observed starting around the end of radiation treatment to the submental muscles. These findings support further research to study the mechanism generating this pathophysiologic deficit, which will be important for developing targeted interventions to prevent or treat the onset of radiation-induced dysphagia. More work is also needed to characterize the changes in tongue kinematics affected and to determine if prophylactic rehabilitation exercises focused on tongue movement/strength during radiation could prevent these outcomes.

## Supporting information

S1 Table Licking data per time point for each treatment. (PDF)

S2 Table Biting data per time point for each treatment. (PDF)
