# Supplementary material for: Acute effects of radiation treatment to submental muscles on burrowing and swallowing behaviors in a rat model
Source: PLoS One. 2022 May 13;17(5):e0268457. doi: 10.1371/journal.pone.0268457 (PMC9106154; doi:10.1371/journal.pone.0268457)
Supplement: S1 Table — (PDF) [file pone.0268457.s001.pdf]

S1: Licking data per time point for each treatment.

Treatment	Time Point	Lick Freq	ILI	CD	Total Clusters	Total Licks
Radiation	Baseline	5.23	0.20	0.08	19	1379
		5.44	0.19	0.09	24	901
		5.50	0.18	0.09	12	1279
		5.75	0.18	0.09	15	1640
		5.47	0.18	0.09	9	1319
		5.19	0.19	0.09	6	1398
		5.75	0.19	0.08	17	1459
	1 week	5.23	0.22	0.08	27	1245
		5.37	0.21	0.08	17	1320
		5.43	0.19	0.09	16	1328
		5.34	0.19	0.08	20	1324
		5.49	0.19	0.08	13	1361
		4.84	0.22	0.08	25	970
		5.59	0.18	0.08	7	1380
	3 weeks	5.04	0.21	0.08	18	1117
		5.50	0.18	0.08	10	1170
		6.09	0.17	0.08	22	1029
		5.59	0.18	0.09	17	1359
		5.09	0.21	0.07	27	711
		4.82	0.22	0.07	30	1048
		5.54	0.19	0.09	11	1293
	4 weeks	4.83	0.22	0.08	20	1110
		5.34	0.19	0.08	21	1147
		5.40	0.19	0.09	18	1189
		5.25	0.19	0.08	21	1211
		5.50	0.20	0.06	40	758
		5.04	0.21	0.08	15	1219
		4.82	0.22	0.08	23	1084
	5 weeks	4.61	0.23	0.09	25	891
		5.50	0.19	0.08	30	1044
		5.33	0.20	0.08	15	861
		4.71	0.22	0.09	17	1046
		5.02	0.20	0.08	18	1006
		4.92	0.21	0.08	15	983
		5.00	0.21	0.09	19	1028
	6 weeks	5.00	0.23	0.08	19	1014
		5.22	0.20	0.09	17	1294
		5.54	0.19	0.09	15	980
		5.32	0.19	0.09	17	1045
		4.88	0.21	0.06	19	1033
		4.51	0.24	0.07	31	745
		5.71	0.18	0.08	14	1184

Treatment	Time Point	Lick Freq	ILI	CD	Total Clusters	Total Licks
Control	Baseline	5.40	0.18	0.08	13	1298
		5.44	0.19	0.08	23	1457
		5.58	0.18	0.08	15	1516
		5.29	0.19	0.09	24	1368
		5.35	0.19	0.08	14	1379
		5.72	0.22	0.09	19	1235
		6.23	0.18	0.09	26	1010
	1 week	5.51	0.19	0.08	18	1165
		5.72	0.18	0.07	21	1194
		5.68	0.18	0.08	22	1463
		5.83	0.18	0.08	35	1056
		5.23	0.19	0.08	17	1217
		5.48	0.19	0.07	12	1270
		5.63	0.18	0.08	13	1519
	3 weeks	5.32	0.20	0.08	20	1080
		5.96	0.18	0.06	29	1039
		5.77	0.21	0.06	34	272
		5.71	0.18	0.07	29	811
		5.44	0.19	0.08	20	1182
		5.21	0.20	0.08	17	1248
		6.05	0.17	0.08	25	1306
	4 weeks	5.66	0.18	0.08	17	1071
		5.85	0.22	0.07	21	993
		5.87	0.18	0.08	30	1292
		5.17	0.21	0.07	40	714
		5.34	0.19	0.08	23	1147
		5.42	0.19	0.08	19	1026
		6.04	0.17	0.08	23	1048
	5 weeks	5.46	0.19	0.09	10	1098
		6.11	0.17	0.07	17	1083
		5.57	0.18	0.08	19	1414
		5.70	0.19	0.08	29	1067
		5.56	0.19	0.08	27	1327
		5.39	0.19	0.09	21	1080
		5.69	0.18	0.08	20	1426
	6 weeks	5.38	0.19	0.09	19	1215
		5.29	0.19	0.08	14	1149
		5.50	0.18	0.08	20	1428
		5.34	0.20	0.08	25	970
		5.21	0.20	0.08	14	1321
		5.52	0.19	0.07	14	1170
		5.64	0.18	0.08	17	1410

ILI = interlick interval, CD= contact duration
