# Supplementary material for: Acute effects of radiation treatment to submental muscles on burrowing and swallowing behaviors in a rat model
Source: PLoS One. 2022 May 13;17(5):e0268457. doi: 10.1371/journal.pone.0268457 (PMC9106154; doi:10.1371/journal.pone.0268457)
Supplement: S2 Table — (PDF) [file pone.0268457.s002.pdf]

S2: Biting data per time point for each treatment.

Treatment	Time Point	Bite Frequency	Intensity	Total Bites	Total Biting Time
Radiation	Baseline	5.17	1.36	1571	377.14
		2.95	1.49	416	239.05
		2.35	1.66	567	442.97
		2.90	1.66	387	216.93
		4.27	1.72	537	186.61
		2.87	2.03	376	213.08
		4.44	1.78	697	265.59
	1 week	5.31	1.58	1513	364.17
		4.42	1.81	429	141.57
		3.58	1.72	594	329.41
		2.82	1.58	428	242.72
		3.42	1.64	522	268.82
		3.19	1.85	419	235.72
		4.46	1.86	692	252.03
	3 weeks	5.21	1.65	1482	474.48
		4.46	1.82	411	136.66
		3.68	1.67	562	240.42
		2.89	1.49	419	239.05
		3.05	1.64	632	406.00
		2.60	1.50	504	413.82
		3.48	2.44	443	268.66
	4 weeks	5.59	1.69	1728	462.43
		3.89	2.10	442	160.61
		4.32	1.77	613	233.57
		3.92	1.49	569	235.14
		4.32	1.47	732	240.13
		3.14	1.47	542	269.60
		4.08	1.84	652	260.72
	5 weeks	4.83	1.38	1709	521.32
		4.35	1.71	515	219.75
		4.64	1.60	713	254.96
		3.52	1.54	586	252.77
		4.13	1.40	776	337.17
		2.57	1.68	621	343.09
		4.49	1.82	935	289.14
	6 weeks	4.97	1.31	1588	432.71
		3.14	1.48	627	301.70
		3.17	1.52	609	420.08
		3.66	1.49	627	255.03
		4.08	1.17	949	330.72
		2.55	1.84	488	346.41
		4.03	1.40	1067	348.00

Treatment	Time Point	Bite Frequency	Intensity	Total Bites	Total Biting Time
Control	Baseline	3.45	1.15	538	203.80
		2.30	2.38	521	441.26
		6.51	1.58	1765	341.76
		2.60	2.01	394	270.93
		3.03	1.70	408	235.36
		5.41	1.74	716	179.36
		4.82	1.74	694	186.32
	1 week	3.60	1.05	511	268.95
		2.33	2.23	471	425.35
		6.01	1.44	1928	465.40
		3.40	2.05	413	230.20
		3.58	1.57	457	226.04
		5.17	1.75	805	247.67
		4.63	2.01	680	233.66
	3 weeks	4.04	1.60	814	332.91
		2.92	2.07	570	396.98
		6.09	1.50	1763	401.47
		3.66	2.22	590	433.00
		3.76	1.56	1178	487.06
		5.77	1.65	802	215.16
		4.25	1.58	568	220.84
	4 weeks	3.05	0.99	458	291.60
		3.22	1.54	529	344.01
		5.60	1.23	1374	477.69
		3.39	2.03	642	284.53
		3.87	1.62	699	314.36
		4.75	1.52	734	217.02
		3.87	1.50	613	290.67
	5 weeks	4.31	1.60	779	330.80
		2.91	1.62	716	386.66
		4.93	1.06	1924	497.14
		2.84	1.92	410	226.25
		3.91	1.28	919	343.70
		5.01	1.68	1120	317.60
		4.65	1.32	1335	401.17
	6 weeks	3.90	1.06	560	236.03
		4.26	1.68	883	333.68
		5.65	1.15	2052	482.51
		4.22	1.53	875	312.39
		4.12	1.23	962	310.87
		4.51	1.21	1102	309.49
		3.72	1.35	955	384.32
